# Sexual Dysfunction in People with Multiple Sclerosis: The Role of Disease Severity, Illness Perception, and Depression

**DOI:** 10.3390/jcm12062215

**Published:** 2023-03-13

**Authors:** Cristiano Scandurra, Laura Rosa, Antonio Carotenuto, Marcello Moccia, Sebastiano Arena, Antonio Ianniello, Agostino Nozzolillo, Mariavittoria Turrini, Lidia Mislin Streito, Gianmarco Abbadessa, Elisabetta Ferraro, Manuela Mattioli, Alessandro Chiodi, Nelson Mauro Maldonato, Simona Bonavita, Marinella Clerico, Cinzia Cordioli, Lucia Moiola, Francesco Patti, Luigi Lavorgna, Massimo Filippi, Giovanna Borriello, Emanuele D’Amico, Carlo Pozzilli, Vincenzo Brescia Morra, Maria Petracca, Roberta Lanzillo

**Affiliations:** 1 Department of Neurosciences, Reproductive Sciences and Dentistry, University of Naples Federico II, 80131 Naples, Italy; 2 Department of Public Health, University of Naples Federico II, 80131 Naples, Italy; 3 Department of Molecular Medicine and Medical Biotechnology, University of Naples Federico II, 80131 Naples, Italy; 4 MS Unit, Federico II University Hospital, 80131 Naples, Italy; 5 Department “G.F. Ingrassia”, MS Center, University of Catania, 95125 Catania, Italy; 6 MS Center, S. Andrea Hospital, Sapienza University of Rome, 00185 Rome, Italy; 7 Neurology Unit, Multiple Sclerosis Center, IRCCS San Raffaele Scientific Institute, 20132 Milan, Italy; 8 Centro Sclerosi Multipla, ASST Spedali Civili di Brescia, Ospedale di Montichiari, 25018 Brescia, Italy; 9 San Luigi Gonzaga Academic Hospital, 10043 Orbassano, Italy; 10 Department of Advanced Medical and Surgical Sciences, University of Campania “Luigi Vanvitelli”, 81100 Naples, Italy; 11 S. Filippo Neri Hospital, 00135 Rome, Italy; 12 NCL-Istituto di Neuroscienze Gruppo Neuromed, 00178 Rome, Italy; 13 Intradepartmental Program of Clinical Psychology, Federico II University Hospital, 80131 Naples, Italy; 14 Vita-Salute San Raffaele University, 20132 Milan, Italy; 15 MS Center, San Pietro Hospital Fatebenefratelli, 00189 Rome, Italy; 16 Department of Human Neurosciences, Sapienza University, 00185 Rome, Italy

**Keywords:** multiple sclerosis, severity, illness perception, depression, sexuality

## Abstract

Despite being a common issue in people with multiple sclerosis (pwMS), sexual dysfunction is still underinvestigated. This work aims to assess the potential determinants of sexual dysfunction in pwMS by considering its relationship with disease severity (in terms of global disability), illness perception, and depressive symptoms. In this multicenter study, 1010 pwMS responded to an online survey. A serial mediation model considering negative illness perception and depressive symptoms as mediators of the relationship between disease severity and sexual dysfunction was conducted using the SPSS PROCESS Macro with bias-corrected bootstrapping (5000 samples). Disease severity exerts an indirect effect on sexual dysfunction via illness perception, both independently and through depressive symptoms. However, the results indicated that illness perception plays a more crucial role in sexual dysfunction in pwMS with mild disability than in pwMS with moderate-severe disability. This study suggests that higher disability increases its magnitude by enhancing negative illness perception, that, in turn, affects sexual dysfunction both directly and through depressive symptoms, especially in pwMS with mild disability. Modulating the effect of illness perception by favoring adaptive coping strategies might represent a valid approach to mitigate sexual dysfunction symptoms in MS.

## 1. Introduction

Multiple sclerosis (MS) is a chronic neurodegenerative disease that affects the motor and cognitive systems, increasing the risk of developing negative mental health outcomes, such as depressive symptoms [1,2,3,4,5]. To this end, previous research has found that approximately 25–50% of people with MS (pwMS) have some form of major depression [6] and that the higher the level of disability, the higher the risk of developing depressive symptoms is [7,8].

However, within the Common Sense Model (CSM) [9], which is rooted in health psychology, pwMS illness perceptions are assigned a predominant role in the pathway to health [10], moving beyond objective factors (i.e., disease severity) as predictors of negative mental health outcomes. According to the CSM, cognitive representations and beliefs that people with a chronic disease develop based on illness stimuli (e.g., symptoms) can influence their illness outcomes. Indeed, people with a chronic disease form commonsense beliefs about their illness (particularly related to identity, consequences, causes, timing, and control) to cope with health threats, and these beliefs drive people’s coping and emotional responses to such threats. To this end, some people with chronic diseases do not develop psychological problems despite a high degree of disability thanks to resilience processes, whereas others develop mental health problems despite a low degree of objective disability [11]. This occurs because people with chronic diseases may develop both negative and positive illness beliefs about their own disease, and this may influence their ability to cope with the disease, leading them to perceive it as threatening or manageable [12]. Thus, within this theoretical framework, negative illness perceptions have been widely demonstrated to act as mediators between disease severity and mental health or quality of life [13,14,15]. A recent extension of the CSM provided a more comprehensive explanations of relationships between representations of health threats, coping responses, and illness outcomes, by stressing the importance of specifying the mediating effect of threat representations in motivating approach- or avoidance-oriented coping strategies or, differentiating illness representations and beliefs, and assessing the moderating role of illness type, personality traits, or emotional representations [16].

Aside from the negative impact on mental health, pwMS often suffer from sexual dysfunction, the causes of which appear to depend on physical impairments, psychological factors, and medication side effects [17]. Although sexual dysfunction is widely recognized in pwMS [18,19,20], it is poorly assessed, underdiagnosed, and undertreated in both research and clinical practice [21].

Interestingly, very few studies using CSM dimensions have considered sexual dysfunction as a potential health outcome, despite the fact that sexual functioning is often affected by chronic disease [22,23]. For example, Daleboudt et al. [24] found that illness perception affects sexual functioning more than disease severity in people with systemic lupus erythematosus. Knowles et al. [25] examined relationships among several variables (e.g., illness perceptions, body image, self-consciousness, sexual health, mental health) in patients with inflammatory bowel disease and found that, among other significant associations, illness perception negatively affected sexual functioning and that this relationship was mediated by depression.

**Figure 1:**
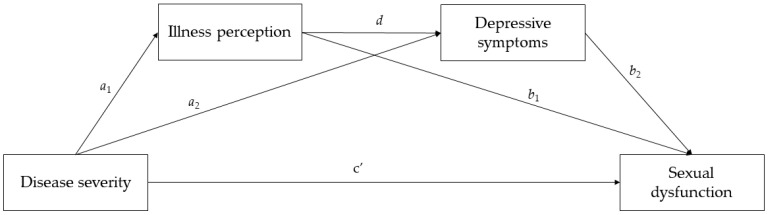
The hypothesized serial multiple mediator model.

To fill the gap in the literature regarding the lack of studies assessing sexual dysfunction in pwMS in the context of CSM, the current study aims to test a serial mediation model in which negative illness perception and depressive symptoms were considered as two potential serial mediators of the relationship between disease severity and sexual dysfunction in a large group of Italian pwMS. Specifically, based on our theoretical background, we expected that higher disease severity (in terms of global disability) would increase negative perceptions of illness, which, in turn, would lead to an increase in depressive symptoms, and that this increased level of depression would possibly affect sexual dysfunction. The hypothesized model is shown in Figure 1.

## 2. Materials and Methods

### 2.1. Procedures and Participants

Data for the current study were collected through a cross-sectional web-based survey conducted from February to July 2021 and uploaded to the European Commission’s official survey management tool (https://ec.europa.eu/eusurvey (accessed on 28 February 2021)).

Participants were reached through the official websites of the participating Italian clinical centers (i.e., SMsocialnetwork.com and webpages of the participating MS centers). Once participants clicked on the link, they were redirected to the first page of the survey, which provided information about the investigators, objectives, study design, benefits, and risks. With the goal of matching data from neurological examinations and self-reports, the survey was not anonymous. Data about MS diagnosis, clinical phenotype (relapsing or progressive form) and disease severity (in terms of objective disability status assessed via the Expanded Disability Status Scale—EDSS) were provided by each participating center at the end of the enrollment period. Specifically, the EDSS was retrieved from the different MS centers retrospectively, and refers to the visit closest to the survey completion in an interval period of ± 3 months. All other information/measures were collected through the online survey. The inclusion criteria were: (1) age ≥ 18 years (Italian age of consent); (2) a confirmed MS diagnosis (by the enrolling center); and (3) Italian language proficiency. Participants were recruited from 11 Italian MS centers, representative of the entire Italian territory.

The study enrollment target was fixed, a priori, at 1000 participants, a population considered large enough to provide meaningful data. The enrollment period was set to six months (from February to July 2021), with a backup strategy to extend the enrollment period in case the enrollment target could not be reached. The enrollment period was therefore closed in September 2021.

The project was approved by the ethical committee of the University of Naples (protocol number: 171/19; date of approval: 12 June 2019), designed in respect of the principles of the Declaration of Helsinki, and conducted following the EU General Data Protection Regulation.

### 2.2. Measures

#### 2.2.1. Socio-Demographic and Clinical Information

We collected the following sociodemographic and clinical variables: referring center, gender, age, sexual activity in the prior six months (yes vs. no), comorbidities that may influence sexuality, i.e., neurologic, pulmonary, cardiovascular, endocrine, and metabolic comorbidities (yes vs. no).

#### 2.2.2. Disease Severity

Disease severity was quantified in terms of objective physical disability, assessed via the Expanded Disability Status Scale (EDSS) [26], the most widely used measure of disability in clinical practice and MS trials [27]. This clinician-report scale score ranges from 0 (normal) to 10 (death due to MS) in 0.5-unit increments. Higher scores on EDSS represent greater severity.

#### 2.2.3. Illness Perceptions

Illness perceptions about one’s MS were assessed using the Italian version of the Brief Illness Perception Questionnaire (BIPQ) [28,29], an eight-item scale measuring the emotional and cognitive representations of illness on an 11-point Likert scale. An example item is “How long do you think your illness will continue?” with response options ranging from 0 (“a very short time”) to 10 (“forever”). The total score is obtained by adding the individual scores for each question and dividing by the number of items. Higher scores reflect a negative subjective perception and indicate a higher perceived threat. The α coefficient for the current sample was 0.72.

#### 2.2.4. Depressive Symptoms

Depressive symptoms were measured using the corresponding subscale from the Neuro-QoL [30], a set of self-reported measures that assesses the health-related quality of life of adults and children with neurological disorders. Specifically, the depressive symptoms short-form is an eight-item scale that investigates negative mood, decrease in positive affect, experience of loss and feelings of hopelessness, cognitive symptoms, negative views of the self, and negative social cognition. Response options range from 1 (“never”) to 5 (“very often”), with higher scores reflecting greater presence and frequency of depressive symptoms. The α coefficient for the current sample was 0.91.

#### 2.2.5. Sexual Dysfunction

Sexual dysfunction was measured using the Italian version of the Multiple Sclerosis Intimacy and Sexuality Questionnaire (MSISQ-19) [18,31], a 19-item scale designed to assess the severity of symptoms of sexual dysfunction in pwMS. The MSISQ-19 assesses three dimensions of sexual dysfunction, classified as primary (i.e., symptoms that result from a neurogenic condition and directly affect sexual functioning, such as orgasmic dysfunction), secondary (i.e., symptoms resulting from a neurogenic disease that indirectly affect sexual functioning, such as fatigue), and tertiary (i.e., emotional, psychological, and social aspects of a neurogenic disease that affect sexual functioning, such as insecurity about one’s sexuality). Response options range from 1 (“never”) to 5 (“always”), with higher scores reflecting greater sexual dysfunction. For statistical parsimony, we used the global score in the current study, which can range from 19 to 95. The α coefficient for the current sample was 0.94.

### 2.3. Statistical Analyses

All statistical analyses were performed using the Statistical Package for the Social Sciences version 27. The significance level for all statistical tests was set at α = 0.05.

Bivariate correlations between study variables (disease severity, illness perception, depressive symptoms, and sexual dysfunction) were calculated using Pearson’s coefficient.

The PROCESS Macro for SPSS (Model 6) [32] was used to test the statistical significance of the direct and serial mediation effects with bias-corrected bootstrapping (5000 samples) and 95% confidence intervals (CIs). Indirect effects were considered significant if the upper (UL) and lower (LL) boundaries of the bias-corrected 95% CIs did not contain zero. As sociodemographic and clinical variables may influence sexual dysfunction [33], we adjusted the model by including potential confounding variables, namely gender (male vs. female), age, sexual activity (yes vs. no), and comorbidities that may influence sexuality (yes vs. no). In order to verify our hypothesis across different levels of disability, we performed a post-hoc analysis, dividing our sample and testing our model independently in patients with mild disability (EDSS ≤ 3.5; *n* = 805) and moderate-severe disability (EDSS > 3.5; *n* = 205).

To avoid problems of multicollinearity, all linear variables were mean centered. In addition, data were also checked for multicollinearity using the variance inflation factors (VIFs). The VIFs were acceptable, ranging from 0.682 to 1.467 [34].

## 3. Results

### 3.1. Participants’ Characteristics

From February to September 2021, 1229 people, self-identifying as MS patients, completed the online survey. After eliminating subjects whose MS diagnosis was not confirmed by the enrolling center, subjects that did not complete the MSISQ-19, did not have availability of EDSS within three months from the date of survey completion, or experienced disability worsening or relapses in the time period between the neurological examination and the survey completion, the final study population included 1010 pwMS.

Participants’ age ranged from 18 to 71 (Mean [M] = 40.57; Standard Deviation [SD] = 10.61). Regarding gender identity, 336 (33.3%) self-identified as men and 674 (66.7%) as women. Most participants were sexually active in the past 6 months (*n* = 824; 81.6%), whereas slightly more than a quarter of the sample (*n* = 266; 26.3%) suffered from some form of comorbidity that affected their sexuality (e.g., polycystic ovary, epilepsy, etc.) Finally, most participants suffered from relapsing-remitting MS (87%), and the remainder suffered from progressive MS (13%).

### 3.2. Descriptive Statistics and Bivariate Correlations

Means, standard deviations, and Spearman’s rank order correlations are reported in Table 1. The results showed that all dimensions considered were positively correlated with each other. In particular, disease severity correlated positively with negative illness perceptions, depressive symptoms, and sexual dysfunction, and the latter three variables correlated positively with each other.

### 3.3. Serial Mediation Analysis

**Figure 2:**
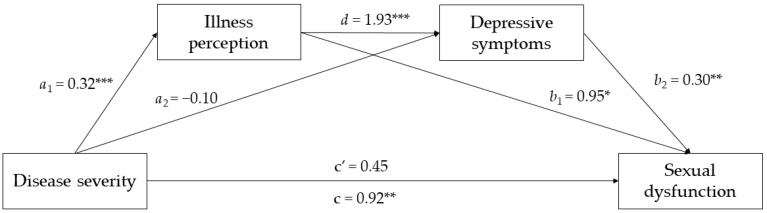
The mediated effects of illness perception and depressive symptoms on the relationship between disease severity and sexual dysfunction. * *p* < 0.05; ** *p* < 0.01; *** *p* < 0.001.

Serial mediation analysis was performed to test whether the association between disease severity and sexual dysfunction was mediated by negative illness perceptions and depressive symptoms, after adjusting for all covariates. The effects of the paths linking disease severity to each mediator and sexual dysfunction are shown in Figure 2.

As shown in Table 2, the results indicated that the first indirect path (disease severity → illness perception → sexual dysfunction) was significant, suggesting that negative illness perception mediated the relationship between disease severity and sexual dysfunction. In contrast, the second indirect path (disease severity → depressive symptoms → sexual dysfunction) was not significant. Instead, the third indirect path (disease severity → illness perception → depressive symptoms → sexual dysfunction), which relates to the main hypothesis of the current study, was significant. This result suggests that higher disease severity increases the magnitude of sexual dysfunction by increasing both negative illness perceptions and depressive symptoms (i.e., hypothesized mediators). Table 2 also shows the results on each pathway.

However, although the total effect of disease severity on sexual dysfunction was significant (*β* = 0.917; *t* = 3.156; 95% *CI* = 0.347, 1.488; *p* = 0.002) and explained 18.3% of the variance in our outcome, this was not true for the direct effect, which was not significant. As suggested by Hair et al. [35], this is a case of indirect-only mediation, where the indirect effect is significant but not the direct effect, suggesting that there is complete mediation.

Of the control variables, only age (i.e., being older; *β* = 0.12; *p* = 0.015) and the presence of comorbidities that may affect sexuality (*β* = 2.377; *p* = 0.042) proved to be associated with higher levels of sexual dysfunction.

As per our subgroup analysis, we found that disease severity was significantly associated with illness perception in pwMS with mild disability (*β* = 0.287; *t* = 6.013; 95% *CI* = 0.193, 0.381; *p* < 0.001) and that both the direct effect (*β* = 1.511; *t* = 2.587; 95% *CI* = 0.364, 2.657; *p* < 0.010) and the total effect (*β* = 2.100; *t* = 3.621; 95% *CI* = 0.962, 3.239; *p* < 0.001) were statistically significant, explaining 17% of the variance in our outcome. In addition, the first indirect path (disease severity → illness perception → sexual dysfunction; *β* = 0.307; 95% *CI* = 0.045, 0.595) and the third indirect path (disease severity → illness perception → depressive symptoms → sexual dysfunction; *β* = 0.168; 95% *CI* = 0.065, 0.296) were significant, whereas the second indirect path was not (disease severity → depressive symptoms → sexual dysfunction; *β* = 0.115; 95% *CI* = −0.045, 0.292). In contrast, we found that for pwMS with moderate-severe disability, the only significant association was between disease severity and depressive symptoms (*β* = 0.954; *t* = 1.980; 95% *CI* = 0.004, 1.904; *p* = 0.049), whereas all other direct and indirect associations were not statistically significant. These results indicate that illness perception plays a more crucial role in sexual dysfunction in pwMS with mild disability than in pwMS with moderate-severe disability.

## 4. Discussion

In this study, we explored the role of illness perception and depressive symptoms as mediators in the relationship between disease severity (objective disability) and sexual dysfunction in a large sample of Italian pwMS. Our results disclosed the role of illness perception as relevant mediator, and suggest that its influence on sexual dysfunction is exerted not only directly, but also indirectly, through depressive symptoms.

A recent systematic review [36] underlined the existence of a moderate effect size when analyzing the relationship between illness perceptions and outcomes (either psychological, physical, related to illness management or socioeconomic aspects) in MS, with “positive” perceptions (e.g., stronger beliefs of control) related to better outcomes, and “negative” perceptions (e.g., attribution of negative consequences to the disease) related to worse outcomes. So far however, to the best of our knowledge, only two studies have explored the possible mediational role of illness perceptions in MS, in the relationship between either depression and quality of life [37], or perceived MS-related physical condition and distrust towards treatment efficacy [38]. Here, for the first time, we demonstrated that illness perception acts as significant mediator not only when assessing the relationship between self-perceived conditions (depression/physical status) and self-perceived outcomes (quality of life/treatment distrust), but also when evaluating the relationship between objective disease status (physical disability) and self-perceived outcomes (sexual dysfunction). However, our results suggest that illness perception mediates the relationship between disease severity and sexual dysfunction in pwMS, and such findings seem driven by subjects with mild disability, likely indicating that in pwMS with moderate-severe disabilities, the perceptions associated with their disease are less salient than the objective condition.

Along the same line, objective disability seems to affect MSISQ-19 scores only in pwMS with mild disability. As the EDSS score expresses the presence of deficits in relevant functional systems (pyramidal, cerebellar, brainstem, sensory, bowel and bladder, visual, mental), higher EDSS scores would likely result in higher levels of sexual dysfunction, driven by higher scores in both the primary and secondary sexual dysfunction domains. However, our results seem to suggest that this is more likely when the disability is not severe. In this case, such a condition might be more likely to affect mental health (e.g., depressive symptoms) than sexual dysfunction. It may be plausible to hypothesize that the failure to examine potential differences between disability levels has led to inconsistent results in the associations between physical disability (assessed via the EDSS) and sexual dysfunction (assessed via the MSISQ-19 scores), where the EDSS was either identified as the only independent risk factor in multivariate regression models [39] or the role of the EDSS was prominent in univariate analysis but weakened in multivariate regression analysis in the presence of depression [40].

Finally, a few words on the role of depression. As per the severity of physical disability, also the impact of depressive symptoms on MSISQ-19 scores is uncertain based on current evidence [39,40]. This variability might be ascribed to differences in the tools used for the assessment of depressive symptoms or to differences in the sample clinical-demographic features. However, none of the previous investigations has considered the possibility that depressive symptoms might be the reflection of a negative illness perception and thus act not as an independent factor but rather as a mediator in this relationship. To further complicate this issue, the relationship between illness perception and depressive symptoms might not be unidirectional, as a recent study has reported illness perception as a mediator of depression impact on the quality of life in MS [37].

Our study is not without limitations. First, we did not investigate the role of illness perception and depression on the relationship between cognitive disability and sexual dysfunction. Although such analysis would have been of interest, we focused on physical disability, as no data on objective cognitive assessment were available for the enrolled population. Furthermore, the cross-sectional nature of our study did not allow conclusive inferences to be made about the temporality and causality of the associations assessed. Future studies should implement a longitudinal design to discern the cause–effect relationships between sexual dysfunction, disease severity, illness perception, and depression. More in general, our investigation suffers from all the known limitations of online surveys (i.e., selection bias and self-reporting of sexual dysfunction). In particular, the selection bias and the fact that data were collected in the first half of 2021 (i.e., during the COVID-19 pandemic lockdown, which had major evidenced negative impacts on the general population and especially in pwMS) might limit the generalizability of our findings. Additionally, due to power limitation, we did not assess the presence of differences in the mediation analysis between relapsing and progressive MS. Regarding the EDSS, we asked the referring centers to exclude patients experiencing disability worsening or relapses in the time period between the neurological examination and the survey completion, but we cannot exclude that, in a minority of cases, the EDSS score entered in the analysis might not correspond to the patient’s status at the time of the survey completion. Although our findings were not confirmed in patients with moderate-severe disability, our investigation in this group was likely underpowered and we cannot exclude that further explorations in larger samples might provide different results. Finally, we did not explore the specific etiology of sexual dysfunction (i.e., the presence of lower spinal cord lesions), as this was beyond the scope of our investigation, which aimed to explore the impact of global objective disability, perceived disability, and depression on sexual dysfunction regardless of its specific cause (primary, secondary, or tertiary).

Notwithstanding its limitations, our study has significant strengths in terms of clinical implications. Namely, our results showed that both illness perceptions and depressive symptoms can influence sexual dysfunction in pwMS, particularly when the disability is not severe, suggesting that helping pwMS process these dimensions may promote sexual health and well-being. Because sexual dysfunction is known to be inadequately addressed by clinicians working with pwMS [41], clinicians should first integrate sexual dysfunction assessment into their clinical approach [42]. In the case of sexual functioning impairment and after biological causes have been ruled out, clinicians should assess the potential role of illness perceptions and depressive symptoms in determining this impairment. This can be done through psychological interventions that focus on assessing illness beliefs and associated coping strategies to explore alternative and more functional coping strategies [43,44,45].

## 5. Conclusions

Our findings suggest that the impact of objective physical disability on sexual dysfunction in MS is mediated by negative illness perception, both directly and through depressive symptoms. Modulating the effect of illness perception by favoring adaptive coping strategies might represent a valid strategy to mitigate sexual dysfunction symptoms in MS.

## Figures and Tables

**Table 1:** Spearman’s correlations between disease severity, illness perception, depressive symptoms, and sexual dysfunction.

	1	2	3	4	M ± SD or Mdn (Range)
1. EDSS	−				2 (0–8.5)
2. Illness perception	0.38 ***	−			5.33 ± 1.44
3. Depressive symptoms	0.16 ***	0.45 ***	−		13.63 ± 6.77
4. Sexual dysfunction	0.14 ***	0.19 ***	0.19 ***	−	36.03 ± 15.06

EDSS = Expanded Disability Status Scale; M = Mean; SD = Standard Deviation; Mdn = Median. *** *p* < 0.001.

**Table 2:** Serial mediation analysis results.

	*β*	BootSE	*t*	*p*	BootLLCI	BootULCI
Outcome: Illness perception						
Disease severity	0.317	0.026	12.214	<0.001	0.266	0.368
Outcome: Depressive symptoms						
Disease severity	−0.100	0.121	−0.826	0.409	−0.338	0.138
Illness perception	1.929	0.146	13.224	<0.001	1.643	2.215
Outcome: Sexual dysfunction						
Disease severity (direct effect)	0.450	0.310	1.453	0.147	−0.158	1.058
Illness perception	0.995	0.408	2.441	0.015	0.195	1.795
Depressive symptoms	0.298	0.086	3.463	0.001	0.129	0.467
Indirect effects						
Disease severity → illness perception → sexual dysfunction	0.315	0.141	−	−	0.039	0.590
Disease severity → depressive symptoms → sexual dysfunction	−0.030	0.039	−	−	−0.116	0.042
Disease severity → illness perception → depressive symptoms → sexual dysfunction	0.182	0.060	−	−	0.074	0.309

*β* = standardized regression coefficient; BootSE = Bootstrap Standard Error; *t* = *t*-value; *p* = *p*-value; BootLLCI = Lower Bootstrap Confidence Interval; BootULCI = Upper Bootstrap Confidence Interval. The analysis was controlled for gender, age, sexual activity, and comorbidities.

## Data Availability

Data will be made available upon reasonable request to the corresponding author.
